# Application of Endophytic *Pseudomonas fluorescens* and a Bacterial Consortium to *Brassica napus* Can Increase Plant Height and Biomass under Greenhouse and Field Conditions

**DOI:** 10.3389/fpls.2017.02193

**Published:** 2017-12-22

**Authors:** Richard D. Lally, Paul Galbally, António S. Moreira, John Spink, David Ryan, Kieran J. Germaine, David N. Dowling

**Affiliations:** ^1^EnviroCORE, The Dargan Research and Innovation Centre, Department of Science and Health, Institute of Technology, Carlow, Carlow, Ireland; ^2^Oak Park Crops Research Centre, Teagasc, Carlow, Ireland; ^3^Dundalk Institute of Technology, Dundalk, Ireland

**Keywords:** endophytes, oilseed rape, PGPR bacteria, biomass, yield, field experiments

## Abstract

Plant associated bacteria with plant growth promotion (PGP) properties have been proposed for use as environmentally friendly biofertilizers for sustainable agriculture; however, analysis of their efficacy in the field is often limited. In this study, greenhouse and field trials were carried out using individual endophytic *Pseudomonas fluorescens* strains, the well characterized rhizospheric *P. fluorescens* F113 and an endophytic microbial consortium of 10 different strains. These bacteria had been previously characterized with respect to their PGP properties *in vitro* and had been shown to harbor a range of traits associated with PGP including siderophore production, 1-aminocyclopropane-1-carboxylic acid (ACC) deaminase activity, and inorganic phosphate solubilization. In greenhouse experiments individual strains tagged with gfp and Km^r^ were applied to *Brassica napus* as a seed coat and were shown to effectively colonize the rhizosphere and root of *B. napus* and in addition they demonstrated a significant increase in plant biomass compared with the non-inoculated control. In the field experiment, the bacteria (individual and consortium) were spray inoculated to winter oilseed rape *B. napus* var. Compass which was grown under standard North Western European agronomic conditions. Analysis of the data provides evidence that the application of the live bacterial biofertilizers can enhance aspects of crop development in *B. napus* at field scale. The field data demonstrated statistically significant increases in crop height, stem/leaf, and pod biomass, particularly, in the case of the consortium inoculated treatment. However, although seed and oil yield were increased in the field in response to inoculation, these data were not statistically significant under the experimental conditions tested. Future field trials will investigate the effectiveness of the inoculants under different agronomic conditions.

## Introduction

The world’s population is expected to increase to 9 billion by 2050 (Tilman et al., 2011) and will require greater levels of crop production to meet the increase in demand for food (Godfray et al., 2010). In addition, world energy supply remains highly dependent on non-renewable resources. The potential of bioenergy crops, in conjunction with other renewable energy sources, has received a great deal of interest over the last decade (Hill et al., 2006). However, one of the main concerns regarding bioenergy crops is the intensive agricultural practices associated with their production (Germaine et al., 2010) and the associated negative effect on the environment. Impacts include a loss in biodiversity (Geiger et al., 2010), the contamination of water ways causing eutrophication, loss of aquatic biodiversity and drinking water sources (Camargo and Alonso, 2006), and causing destruction to soil through degradation over time (Oldeman, 1992). *Brassica napus* L. (oilseed rape) is an important global crop that is a major source of vegetable oil for human consumption (USDA-FAS, 2015) as well as a source of oil for producing biodiesel. In addition, winter oilseed rape is used as a break crop in cereal production and has been shown to reduce “take-all” fungal disease in wheat when used as part of a crop rotation (Angus et al., 1991; Hilton et al., 2013).

Microorganisms play a key role in the health and development of crops (Tikhonovich and Provorov, 2011; Cory and Franklin, 2012) and the relationship between rhizobacteria and endophytes with their plant hosts has been reviewed extensively (Ryan et al., 2008; Hayat et al., 2010; Mercado-Blanco and Lugtenberg, 2014). Such plant growth promoting rhizobacteria (PGPR) have considerable potential as biological inoculants in sustainable agriculture (Saharan and Nehra, 2011; Glick, 2012; Sivasakthi et al., 2014). Plants that are inoculated with PGPR benefit from the resulting plant–microbe interaction as the bacteria contribute to plant growth and health by multiple mechanisms including nitrogen fixation, synthesis of phytohormones, modulation of plant ethylene levels, solubilization of unavailable soil phosphate and suppression of pathogens through niche exclusion, and the production of anti-microbial metabolites (Fuentes-Ramirez and Caballero-Mellado, 2005; Franche et al., 2009; Babalola, 2010; Compant et al., 2010).

A collection of plant associated bacteria from various plant hosts (*Miscanthus* × *giganteus*, *B. napus*, and *Iris pseudacorus*) have been isolated and partially characterized (Otieno et al., 2013). Strains were isolated following standard procedures from rhizosphere and internal plant tissues including root, leaves, and stem (Germaine et al., 2004, 2009; Otieno et al., 2013). Many of these strains have multiple plant growth promotion (PGP) characteristics including siderophore production, 1-aminocyclopropane-1-carboxylic acid (ACC) deaminase activity (for the regulation of plant stress hormone ethylene caused by abiotic stress conditions), phosphate solubilization, and in some cases biocontrol against fungal plant pathogens.

Of particular interest are three bacterial strains isolated from *Miscanthus* × *giganteus* and a “Mastermix consortium” (MM consortium) of these strains combined with seven other isolated PGP microorganisms. These three strains are *P. fluorescens* L228, L111, and L321 which have been shown to have PGP potential *in vitro* (Brennan, 2010; Otieno et al., 2013, 2015) and *in silico* following genome sequence analysis (Moreira et al., 2016). We also included in this study *P. fluorescens* F113 a well-characterized biocontrol and PGPR strain that was originally isolated in Ireland from *Beta vulgaris* (L) (sugar beet) (Shanahan et al., 1992; Redondo-Nieto et al., 2013). This serves as a useful reference strain in our study. *P. fluorescens* are members of the Class gammaproteobacteria and are a very diverse complex (Garrido-Sanz et al., 2016). *Pseudomonas* spp. are important bacteria in agriculture and have been shown to promote growth, protect plants from pathogens and herbivores (Adesemoye and Egamberdieva, 2013; Sivasakthi et al., 2014), play a role in phytoremediation (Germaine et al., 2009; Glick, 2012), and are a part of the core microbiome of many plants.

The aim of this study is to investigate the potential of these bacteria as biofertilizers in greenhouse and field conditions to determine if they induce PGP of oilseed rape, an important biofuel, food, and break crop in Europe and globally. The colonization potential of each of these strains was examined on oilseed rape using *gfp* marked derivatives under greenhouse conditions to determine the interaction of each strain in the rhizosphere, root, leaf, and PGP effects. Finally, a field trial was undertaken to investigate the impact of these inoculants under standard Irish agronomic conditions for oilseed rape on crop growth and biomass parameters.

## Materials and Methods

### Greenhouse Experiments

Green fluorescent protein (gfp) expressing; kanamycin-resistant marker (Km^r^) derivatives of *P. fluorescens* L321, L228, L111, and F113 (**Table 1**) were constructed as described in Germaine et al. (2004) through biparental mating with *E. coli* S17.1 λpir pUTmTn5gusA-pgfp21 (Xi et al., 1999). The greenhouse experiment consisted of four bacterial treatments (F113*gfp*:*km^r^*, L321*gfp*:*km^r^*, L111*gfp*:*km^r^*, and L228*gfp*:*km^r^*) and a control (no bacterial inoculation). *B. napus* var Compass seeds were surface sterilized with 95% ethanol and dried aseptically, in a laminar cabinet. The seeds were then coated with an alginate preparation containing the PGP strains essentially as described by Power et al. (2011). Alginate coatings were prepared using a solution of 5% alginate, 10% skimmed milk and bacterial culture, made to a final concentration of 10^6^ CFUs ml^-1^ to give a concentration of bacteria of approximately 10^5^ to 10^6^ g^-1^ seed. The alginate solution was dropped over the seeds, using a Pasteur pipette, to coat the seeds and the seeds were added to a 2% calcium chloride solution. The coated solidified seeds were washed twice in sterile water to remove the excess salt and then transferred to a sterile petri dish. The negative control was seed coated with alginate that had no cultures present. Seeds were transferred into plastic horticultural pots containing 500 g of topsoil (clay loam, pH 5.5–6.0, 5% soil organic matter). All soil was mixed prior to the experiment and randomly divided into pots. This ensured nutrient uniformity across the experiment. Plants were watered equally two to three times weekly (typically 50–100 ml of water was added to each pot). There were no added nutrients applied to the pots during the greenhouse experiment.

**Table 1:** List of strains used in this study; origin and plant growth promoting characteristics.

Strain	Identification	Plant of origin	Isolated from	Origin	Plant growth promotion characteristics^a^	Reference
L111^∗^	*Pseudomonas fluorescens*	*Miscanthus* × *giganteus*	Leaf	Oak Park, Co. Carlow, Ireland	Sid, PS, ACC	Otieno et al., 2013; Moreira et al., 2016
L117	*Pseudomonas* sp.	*Miscanthus* × *giganteus*	Leaf	Oak Park, Co. Carlow, Ireland	Sid, BC, PS, ACC	Otieno et al., 2013
L130	*Pseudomonas* sp.	*Miscanthus* × *giganteus*	Leaf	Oak Park, Co. Carlow, Ireland	BC, PS	Otieno et al., 2013
L132	*Pseudomonas* sp.	*Miscanthus* × *giganteus*	Leaf	Oak Park, Co. Carlow, Ireland	Sid, BC, PS	Otieno et al., 2013
L228^∗^	*Pseudomonas fluorescens*	*Miscanthus* × *giganteus*	Leaf	Oak Park, Co. Carlow, Ireland	Sid, BC, PS	Otieno et al., 2013; Moreira et al., 2016
L321^∗^	*Pseudomonas fluorescens*	*Miscanthus* × *giganteus*	Leaf	Oak Park, Co. Carlow, Ireland	PS, ACC	Otieno et al., 2013; Moreira et al., 2016
R324	*Serratia* sp.	*Miscanthus* × *giganteus*	Rhizosphere	Oak Park, Co. Carlow, Ireland	PS	Otieno et al., 2013
R232	*Enterobacter* sp.	*Miscanthus* × *giganteus*	Rhizosphere	Oak Park, Co. Carlow, Ireland	PS	Otieno et al., 2013
S120	*Serratia* sp.	*Miscanthus* × *giganteus*	Stem	Oak Park, Co. Carlow, Ireland	PS	Otieno et al., 2013
Rt03	*Pseudomonas* sp.	*Brassica napus*	Root	Oak Park, Co. Carlow, Ireland	PS	Otieno et al., 2013
F113^∗^	*Pseudomonas fluorescens*	*Beta vulgaris*	Rhizosphere	Fota, Co. Cork, Ireland	Sid, PS, BC, ACC	Shanahan et al., 1992; Redondo-Nieto et al., 2013

*^a^Sid, siderophore production; PS, phosphate solubilization capability; BC, biocontrol activity against Pythium ultimum or Fusarium oxysporum; and ACC, 1-aminocyclopropane-1-carboxylic acid deaminase activity. ^∗^Strains used as single inoculants. All strains with the exception of F113 were used as the MM consortia application.*

Greenhouse growing conditions were as follows: light cycle of 16 h light and 8 h dark phase, with a mean light phase temperature of 30°C and dark phase temperature of 24°C. Per treatment there were 15 pots at the start of the experiment. Three pots were randomly selected for sampling at each time point for CFU analysis (*n* = 3), there was one seed planted per pot.

Greenhouse plant samples were harvested from each treatment (*n* = 3) after 2, 5, 13, 18, and 24 weeks to assess the colonization of the strains on the plants. Rhizosphere, root, and surface sterilized leaf samples were assessed to determine the CFUs in each plant compartment. Surface leaf sterilization was carried out to determine the strains endophytic capability by the method described in Germaine et al. (2004). This was carried out by washing the plant sample with sterilized deionized water for 30 s, followed by a wash in 10% sodium hypochlorite for 1 min, then a final wash in 95% ethanol for 20 s. Samples were then rinsed in sterile deionized H_2_O and 100 μl of the rinse water was spread plated in triplicate on Nutrient Agar “E” (Lab M), to validate the surface sterilization procedure.

### Microbial Counts in Root, Rhizosphere, and Leaf Tissue

Colony forming units per gram of sample were determined using a sucrose glutamine agar (SGA) (Lally, 2016) which promotes the production of fluorescent siderophore, supplemented with kanamycin to select for kanamycin resistance. This media was prepared with H_2_O in 1 l bottles using 20 g sucrose, 2 g glutamine, 1 g K_2_HPO_4_, and 15 g Technical Agar No. 1. After autoclaving, 5 ml of 10% MgSO_4_ was added per liter of media. Kanamycin was added to a final concentration of 50 μg ml^-1^ and cycloheximide to 32.5 μg ml^-1^. Plant tissue was homogenized aseptically with a pestle and mortar and bacterial CFUs were enumerated by dilution and plate counts (Miles and Misra, 1938). Homogenized samples (0.1 g per replicate) of rhizosphere, root, and leaves were added to 900 μl of 14 strength Ringer’s solution. Samples were vortexed for 1 min and serially diluted 1:10 until a 10^-7^ dilution was reached. Aliquots of 20 μl were plated from each dilution onto agar plates in quintuplicate. Plates were then dried in a laminar airflow cabinet, inverted, and then incubated at 30°C. Plates were monitored for 3 days and *P. fluorescens* CFUs were recorded. A number of colonies were randomly selected to examine for *gfp* expression under UV light, using an epifluorescence microscope.

### Dry Plant Weight Following Greenhouse Harvest

At week 24, all plant samples were harvested from the remaining pots. The plant samples were weighed to determine fresh weight (FW). The samples were cut and dried in foil containers for 48 h at 70°C. The samples were then weighed to determine the dry weight (DW) and moisture content. Plant length was determined by measuring the length of the above ground plant material.

### Epifluorescence Microscopy

Epifluorescent microscopy was conducted at the final time point of the experiment. Three plants were used for each treatment (*n* = 3). Root samples were taken at week 24 and mounted for cryostat sectioning. Samples were submerged in PolyFreeze (Sigma-Aldrich), frozen using a cryostat instrument (Leica, CM 1510 S) at -20°C, and sliced to 3 μm longitudinally. Specimens were mounted on glass slides, stained with acridine orange to aid the visualization of the green fluorescent protein expressing isolates, and covered with glass cover slides. Each sample was examined at ×400 magnification and ×1000 using immersion oil under blue light (395 nm) using a Nikon eclipse 8i epi-fluorescent microscope equipped with a 100-W mercury short arc photo-optic lamp. The image was then exported as a PDF and converted to a tiff image using GIMP image software (Germaine et al., 2009).

### Field Trial Setup

The field trial was carried out at Teagasc Crop Research Centre, Oak Park, Carlow, Ireland. The variety of *B. napus* “Compass” was chosen for the use in this trial. It was sown in Oak Park Carlow (coordinates N 52° 51′ 59.41″, W 6° 55′ 25.306″). The soil was a loam (20% clay, 37% silt, and 43% sand) with 4.8% organic matter. The previous crop was winter barley, following which the straw was baled the land ploughed, power harrowed, drilled, and rolled.

The field trial was sown in September 2012 and was monitored until harvest in August 2013. The trial was set up as a randomized complete block design and included six blocks. Each block contained six individual treatments (F113, L228, L321, L111, “MM,” and a negative control). There were six replicates per treatment in the field investigation. Each block contained one replicate. These treatments were replicated throughout each block using randomized allocation for each treatment. The treatment plots were 4 m in width × 23 m in length, a total plot area of 92 m^2^, with 0.5 m spacing between each treatment plot and 1 m spacing between each block. The seed was sown at a rate of 60 seeds/m^2^. Post-germination, the trial received three separate spray applications of the bacteria during the period of early crop development. Crops received normal nutrient fertilizer application of nitrogen, phosphorous, and potassium [240 kg/ha N, 35 kg/ha P, and 65 kg/ha K applied in two applications (30% in late February 70% in late March)]. One spray application of Proline fungicide (Bayer Crop Science Ltd., Cambridge, United Kingdom) was applied as a foliar application to control seasonal disease pressures, it was applied during pod development post flowering 40 weeks into the trial and was applied equally on all blocks. Supplementary Figures 1–3 illustrates the field trial and sampling.

### Preparation and Application of Treatments

There were five live inoculant and one control treatments used in the field trial. These were nutrient broth (LabM Limited, Lancashire, United Kingdom) grown inocula of *P. fluorescens* F113, L228, L321, L111, respectively, and the MM consortium of 10 strains S120, Rt03, R324, L232, L132, L117, L130 (**Table 1**) which also included L228, L321, and L111. For individual strain treatments 100 μl of stock culture was added to the sterile media prepared in two 1 l Duran bottles (1 l in each) and incubated by shaking at 100 rpm at a temperature of 30°C for 24°h. For MM consortium preparation, 100 μl of each strain was taken from culture stocks and added to 200 ml of sterile media, incubated as above and then pooled into a 2 l volume. Two liters of inoculum was sprayed directly onto the soil in each treatment plot using portable manual pump pressurized sprayers (equivalent to 217 l/ha). The concentration of the inoculum was approximately 10^6^ CFU ml^-1^ equivalent to an application rate of approximately 2 × 10^7^ CFU m^-2^. It was applied at this concentration three times; 3 weeks after sowing, 12 weeks after sowing, and 28 weeks after sowing. A control dead inoculum (F113 culture autoclaved at 121°C for 15 min) was applied at the same rate (2 l/plot) to each control plot as a negative control.

### Sampling of the Field Trial for Plant Biomass Analysis

The crop was sampled using a set square (0.5 m^2^). Each plot was split randomly into sub-samples, eight plants per plot were used to measure each parameter. Samples were analyzed by block. Destructive samples from the trial were collected on three separate occasions, early development (3 months), mid-development (7 months), and pre-harvest (9 months). All seed and yield analysis were conducted post-harvest. Seed samples were subsamples of the total harvested seed, collected from the central 2.5 m strip of each plot, this was collected by a mechanical harvester. Bags of seed were stored at room temperature until analyzed. Four plots were removed from the analysis due to weed influx at the border strip of four blocks. Sample size is presented in **Table 2**.

**Table 2:** Summary statistics from the field trial of *B. napus* inoculated with plant growth promoting *Pseudomonas* and a bacterial consortium (MM).

Treatment	Sample size (*n*)	Average plant biomass (g) ± standard deviation	*P*-value	% Greater than the control	Average plant length (cm) ± standard deviation	*P*-value	% Greater than the control
F113	6	41.53 ± 2.23	0.245	14.12	154.04 ± 6.29	0.454	3.27
L228	5	41.59 ± 5.48	0.279	14.27	154.76 ± 10.74	0.347	3.74
L111	6	40.37 ± 6.33	0.514	11.3	153.00 ± 3.89	0.703	2.6
L321	5	40.61 ± 5.74	0.502	11.89	158.23 ± 4.09^∗^	0.018^∗^	5.99^∗^
MM	5	45.76 ± 5.66^∗∗^	0.003^∗∗^	23.73^∗∗^	158.28 ± 5.05^∗^	0.017^∗^	5.96^∗^
Control	5	36.05 ± 3.63	–	–	149.07 ± 6.81	–	–

*^∗^P < 0.05; ^∗∗^P < 0.01.*

### Dry Weight, Plant Length, and Yield

At the time of the third sampling date the pods were at the late stages of development resulting in the early deciduous phase of the leaf cycle; therefore, no green leaf area measurements were taken at this time point. Plants were gently extracted from the soil to obtain the root (root length refers to the length of the tap root). Plants with incomplete roots were not measured. The plants were individually measured to determine plant root and stem length. The samples were separated into roots, stems/leaves, and pods. Plant characteristics were counted (stems, leaves, and pods), the plants were weighed fresh and then were dried for a minimum of 24 h at 70°C in a forced air oven until DW stabilized. If further drying was required samples were exposed to the same conditions for an additional period of 16–24 h. DW was then recorded.

The thousand seed weights were measured using a Contador Pfeuffer (Pfeuffer Kitzingen, Germany). When 1000 counted seeds had passed into the drop compartment the total weight of the seeds was taken. This was repeated in triplicate for each sample. Total harvested seed was determined by the plot measurement given by the combine harvester at the time of the harvest. Seed oil content was analyzed by adding samples from the seed harvest to a seed oil analyzer (CropScan 1000B NIR, Technology Systems, Australia). Ten random subsamples were removed from the harvest sample and measured for oil and moisture content.

### Statistics

Greenhouse and field experiments were carried out once. Greenhouse data were analyzed using univariate analysis with one-way ANOVA and *post hoc* tested using the Bonferroni test. Field trial data were analyzed as a randomized complete block design using a univariate ANOVA. Significant data were subjected to *post hoc* testing using Tukey’s HSD test. In both cases, results with a *P*-value lower than *P* = 0.05 were considered significant following *post hoc* testing. All statistical analysis was carried out using SPSS statistical software version 20.0 (IBM, 2012).

## Results

### Colonization Dynamics of gfp-Tagged *P. fluorescens* Strains on *B. napus* (Oilseed Rape) in Greenhouse Experiments

Chromosomal mini-Tn::*gfp km*-tagged derivatives of *P. fluorescens* F113, L111, L228, and L321 were used to investigate the colonization dynamics in different plant compartments of *B. napus*. The Km^r^ allows the direct selection of the inoculant strain and the *gfp* marker allows the strains to be visualized in the plant by Epifluorescence Microscopy (see Supplementary Figures 4A,C). Under these experimental conditions, no Km^r^ CFUs were detected in the uninoculated control treatments (data not shown).

**FIGURE 1:**
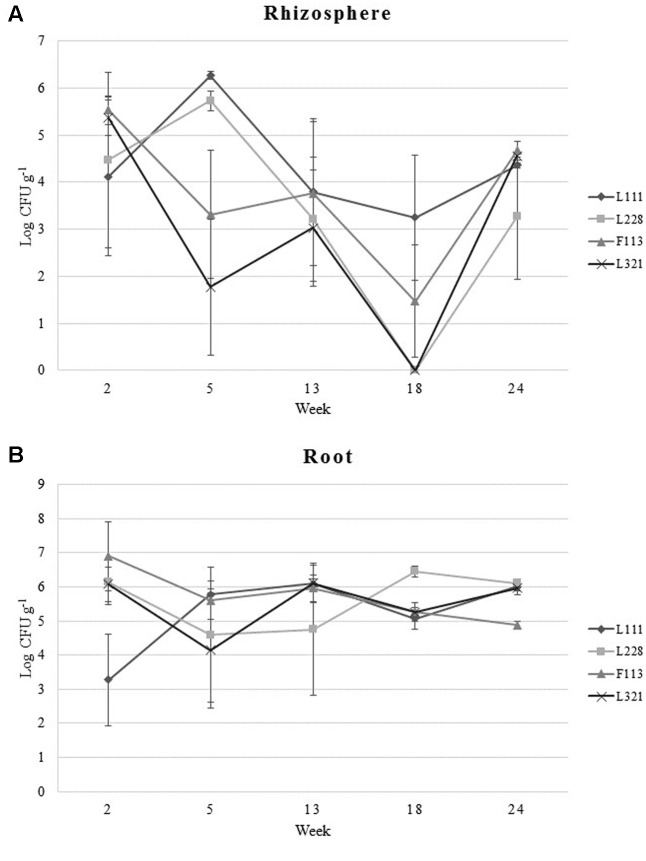
Bacterial colonization of *Brassica napus* by PGP *Pseudomonas* and a bacterial consortium during a 24-week greenhouse trial. **(A)** Bacterial colonization levels of plant rhizosphere. **(B)** Colonization of plant root samples. Data represent the average number of CFUs at each sample time. Error bars represent standard error (±SE).

*Pseudomonas fluorescens* L111, L228, and L321 were found to be effective colonizers of the rhizosphere and they displayed capabilities similar to that of *P. fluorescens* F113*gfp:km^r^*, a proficient rhizosphere colonizer (Villacieros et al., 2005; Rivilla et al., 2013). The strains were capable of colonizing the rhizosphere at levels of 10^3^–10^4^ CFUs g^-1^ (**Figure 1A**) up to 24 weeks post-inoculation. In the rhizosphere at week 2 each strain colonized the rhizosphere at similar levels. At week 5, L111*gfp:km^r^* and L228*gfp:km^r^* had increased in numbers whereas F113*gfp:km^r^* and L321*gfp:km^r^* were seen to decrease from 10^5^ to 10^3^ and 10^2^ CFU g^-1^, respectively. Each of the strains then stabilized at levels of 10^3^ CFU g^-1^. L111 maintained this colonization level at week 18, while F113*gfp:km^r^* showed a decline in numbers. L228*gfp:km^r^* and L321*gfp:km^r^* were undetected at week 18 suggesting an issue with the sampling of these replicates as analysis at week 24 indicated that L111*gfp:km^r^*, F113*gfp:km^r^*, and L321*gfp:km^r^* were present in the rhizosphere at concentrations of 10^4^ CFU g^-1^ and L228*gfp:km^r^* at 10^3^ g^-1^.

Compared with colonization dynamics in the rhizosphere, the root samples had less fluctuations (**Figure 1B**). At week 2, L111*gfp:km^r^* had the lowest population number at 10^3^ CFU g^-1^. The other three strains were present at 10^6^ CFU g^-1^. At week 5 each of the strains maintained uniform population numbers between 10^4^ and 10^5^ CFU g^-1^. At week 13, L111*gfp:km^r^* and L321*gfp:km^r^* reached numbers as high as 10^6^ CFU g^-1^ while L228*gfp:km^r^* remained at the same colonization level as week 5. At week 18, L228*gfp:km^r^* numbers increased to 10^6^ CFU g^-1^ while L111*gfp:km^r^*, F113*gfp:km^r^*, and L321*gfp:km^r^* decreased to 10^5^ CFU g^-1^. At the final sample date, week 24, L321*gfp:km^r^*, L228*gfp:km^r^*, and L111*gfp:km^r^* colonized the roots at levels of 10^6^ CFU g^-1^ and F113*gfp:km^r^* colonized at 10^5^ CFU g^-1^.

Each of the *Pseudomonas* strains were detected in leaf samples after week 2. Isolate L111*gfp:km^r^* was present at levels as low as 10^1^ CFU g^-1^. Strains F113*gfp:km^r^* and L321*gfp:km^r^* were detected at 10^3^ CFU g^-1^ and L228*gfp:km^r^* was observed in the highest numbers at 10^6^ CFU g^-1^. However, there was no further detection of bacterial colonization following subsequent sampling at weeks 5, 13, 18, and 24 (data not presented), indicating that the strains did not colonize the leaf under these experimental conditions.

### Oilseed Rape Inoculated with *P. fluorescens* Strains Show Significant Increases in Plant Biomass in Greenhouse Experiments

Above ground plant biomass was investigated as an indicator of PGP due to inoculant treatment in the greenhouse experiments. The final date of sampling (week 24) was used to obtain samples for above ground biomass analysis (*n* = 3). Statistical analysis was carried out using univariate analysis with one-way ANOVA and *post hoc* tested using the Bonferroni test. There were no differences detected in the moisture levels of the plant samples under the treatment application (data not shown). Supplementary Figures 1–3, 4B show PGP effects due to inoculation with the bacterial treatments.

**FIGURE 2:**
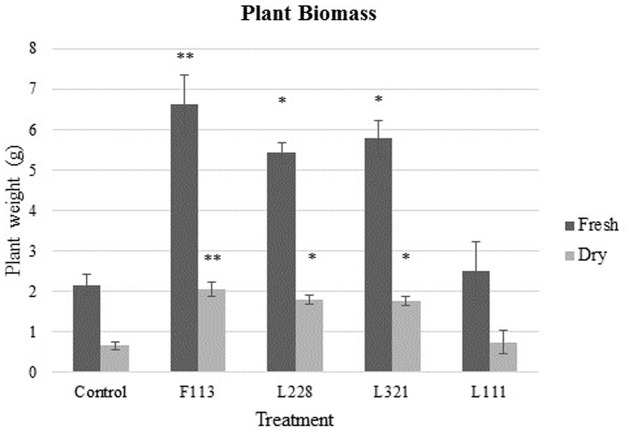
Biomass analysis of *B. napus* in a greenhouse experiment following inoculation by PGP *Pseudomonas* and a bacterial consortium after 24 weeks. Data display above ground (shoots/leaves) plant biomass after 24 weeks colonization with the bacterial strains L111, F113, L321, and L228. Bars represent average value for each data series (*n* = 3). Error bars represent standard error (±SE). ^∗∗^*P* < 0.01; ^∗^*P* < 0.05.

Fresh weight biomass (above ground) was significantly increased (**Figure 2**) in each bacterial treatment except for L111 under the Bonferroni test (F113*gfp*:*km^r^ P* = 0.003, L321*gfp*:*km^r^ P* = 0.013, and L228*gfp*:*km^r^ P* = 0.024). L321*gfp*:*km^r^* showed an average increase of 1.10 g above the control, L228*gfp*:*km^r^* showed an average of 1.13 g greater than the control and F113*gfp*:*km^r^* showed 1.39 g greater than the control. DW biomass analysis also displayed significant increases under Bonferroni test in each treatment except the L111*gfp*:*km^r^* treatment (F113*gfp*:*km^r^ P* = 0.005, L321*gfp*:*km^r^ P* = 0.021, L228*gfp*:*km^r^ P* = 0.018) (**Figure 2**).

### Oilseed Rape Inoculated with *P. fluorescens* L321 and a Bacterial Consortium Show Significant Increases in Plant Length and Biomass in the Field

Following spray inoculation in the field trial, samples were taken at three time points early development (3 months), mid-development (7 months), and pre-harvest (9 months), and analyzed for a number of key agronomic parameters. Data were analyzed statistically. For the first two sampling times, the data obtained showed that there were no statistical differences between any of the treatments and the control (data not shown).

**FIGURE 3:**
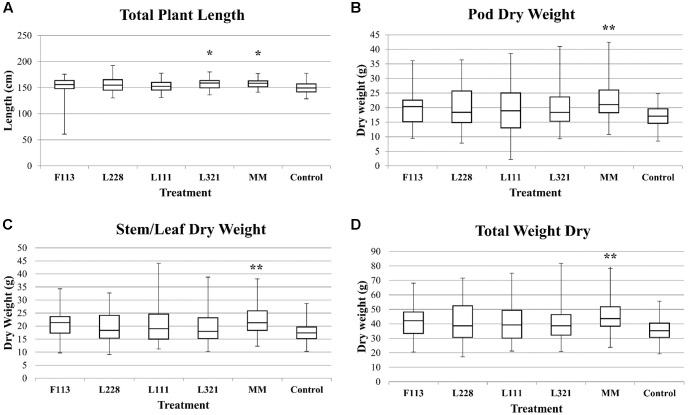
Biomass analysis of *B. napus* in a field experiment following inoculation by PGP *Pseudomonas* and a bacterial consortium at 9 months. Box plots provide a summary of the significant field trial data. These graphs represent the variation within the sample populations. **(A)** The total plant length from the third sample date. **(B–D)** The DW data from the third sampling date. ^∗∗^*P* < 0.01; ^∗^*P* < 0.05.

The third time point (9 months) showed that all inoculated treatments had an increase in biomass compared to the control (**Figure 3**); however, only the MM consortium and *P. fluorescens* L321 were statistically significantly different with respect to the total plant length compared to the control plants. The MM consortium treatment showed a mean difference of 9.20 cm (5.99% increase; *P*-value = 0.012) and *P. fluorescens* L321 showed a mean difference of 9.26 cm (5.96% increase; *P*-value = 0.012) greater than the control (**Figure 3**). Statistically significant results were also observed with respect to dry plant biomass at the third sample date (pre-harvest stage). The control treatment had statistically significantly less biomass in three aspects of plant biomass: stem/leaf, pod, and total plant biomass compared to the MM consortium treatment (**Figure 3**). In the MM treatment, stem/leaf biomass had a mean difference of 4.49 g (22.43% increase; *P*-value = 0.005) greater than the control, the pod weight biomass, a mean difference of 4.93 g (25.11% increase; *P*-value = 0.008), and a total dry biomass mean difference of 9.70 g (23.97% increase; *P*-value = 0.003) compared to the control treatment (**Table 2**). The results gave no indication of negative effects on crop growth as a result of the biofertilizer treatments.

**FIGURE 4:**
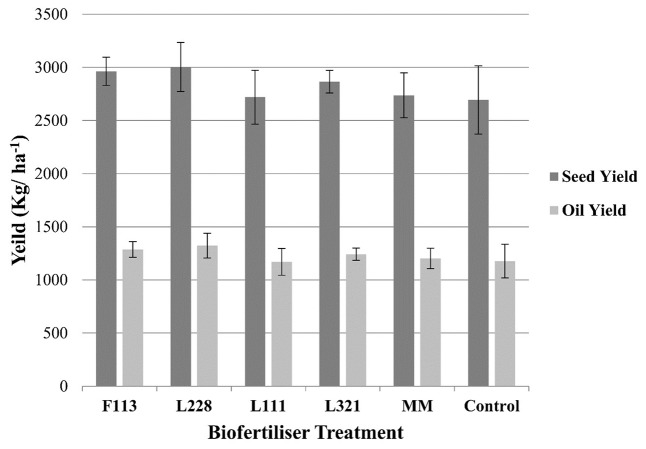
Seed and oil yield output from *B. napus* in a field experiment following inoculation by PGP *Pseudomonas* and a bacterial consortium at harvest. Error bars represent the standard error of the mean (±SE). There were no significant differences found in seed or oil yields.

With respect to the (**Figure 4**) seed weights and oil yield, there was an observable increase in the case of *P. fluorescens* F113 and L228 compared to other treatments and the non-inoculated control; however, these increases were not statistically significant.

## Discussion

In this study, the use of PGP isolates was assessed for their application in sustainable agriculture. Two of the strains *P. fluorescens* L321 and *P. fluorescens* L228 were observed to significantly increase plant biomass in a greenhouse experiment and were efficient colonizers of the rhizosphere and root compartment of *B. napus*, achieving populations as high as the well-characterized rhizobacteria *P. fluorescens* F113 strain.

Each of the strains used in this study are considered plant growth promoting bacteria based on their phenotypic qualities, having been screened for various PGP mechanisms. Their ability to colonize *B. napus* was observed in previous greenhouse trials (Otieno et al., 2013). Strains F113, L228, and L321 produce high levels of gluconic acid and demonstrate high levels of inorganic P solubilization compared to L111 (Otieno et al., 2015) when measured *in vitro* and L321 has previously been shown to increase the growth of pea plants in low phosphate soil (Otieno et al., 2015). This trait may be an important factor in the growth promotion effects observed in the greenhouse study.

Of the five treatments tested in the field trial, two displayed the ability to increase plant growth. Treatments L321 and MM showed the ability to increase plant length by 5.96 and 5.99%, respectively, the MM increased stem/leaf biomass and pod biomass by 23.97% when compared to the control treatment in the trial. In the field experiment two treatments, L321 and the MM, resulted in significantly taller plants, suggesting that direct length promotion may have been due to cell elongation as a result in ACC deaminase activity reducing ethylene levels in the plants and maximizing plant growth (Abeles et al., 1992; Glick et al., 1997; Penrose and Glick, 2001) by ACC deaminase producing strains L111, L117, and L321. This mechanism of PGP usually affects root hair development, resulting in structurally improved rooting systems. In addition there is evidence that strains L111, L228, and L321 can produce the phytohormone indole acetic acid (IAA) (Otieno et al., 2013). As these strains encode genes implicated in the IAA biosynthesis pathway (Moreira et al., 2016) and IAA has been detected by HPLC analysis of culture filtrates (Obermeier and Dowling, Unpublished data). IAA production by these strains may also have contributed to the growth promotion effects observed in both the greenhouse and field experiments.

In the case of the MM treatment multiple strains with a range of PGP traits could improve the chance of exhibiting positive PGP under field conditions. This has been previously suggested as a solution to the inconsistency in results obtained when using single strains as plant growth promoters (Van Veen et al., 1997).

Bacteria exhibiting production of IAA (Idris et al., 2007; Naveed et al., 2014), siderophores (Kloepper et al., 1980; Katiyar and Goel, 2004), phosphate solubilization (Rodríguez and Fraga, 1999; Peix et al., 2001), atmospheric nitrogen fixers (Çakmakç et al., 2001), and ACC deaminase (Mayak et al., 2004; Shaharoona et al., 2006) resulted in plant growth enhancement. In the current study each of the strains used in the MM treatment contained at least three of the PGP traits mentioned above. The data show that the MM consortia treatment was capable of increasing aspects of plant biomass at a field scale, suggesting that multiple mechanisms may have been the key contributors to this finding.

Evidence of the ability of phosphate solubilizing and nitrogen fixing bacterial strains to increase the nutrient uptake and the yield of *Zea mays*, *Triticum aestivum*, *Cicer arietinum*, *Glycine max*, *Lactuca sativa*, *Oryza sativa*, and *Cucumis sativus* has been reported (Adesemoye and Kloepper, 2009; Adesemoye and Egamberdieva, 2013). However, evidence to support the positive effect of microbial biofertilizers on crop yield increase in oilseeds such as *B. napus* is not widely available. However, Kumar et al. (2009) report a significant oil yield increase in a trial of *Sesamum indicum* after inoculation with *Pseudomonas aeruginosa* LES4 and growth promotion of Maize (*Z. mays* L) following inoculation with *Azospirillum* and *Azotobacter* PGPR has also been reported under field conditions (Gholami et al., 2012).

The result of the field trial showed that while, on average, there was an increase in the seed yield due to plant inoculation with these strains (L321:2.65, L228:3.05, and F113:2.65 t/ha) there was no statistically significant difference compared to the seed yields from the control plots (control: 2.45 t/ha). Although these increased yields were not statistically significant, they do in fact represent a significant economic increase for the farmer. In 2016, the market price of rape seed was €357/t (IFA, 2016). The increased yields due to the biofertilizer application would correspond to an average increase of €71.4/ha (with strain L321), €178/ha (with strain F113), and €200/ha (with strain L228). With the cost of application of these biofertilizers ranging from €50 to 100/ha, our results show that the use of these microbes could result in economic benefits for the farmer.

The applications of chemical fertilizers in this field study may have masked our ability to observe significant growth impacts on crop yields. Previous investigations suggest that strategies combining both reduced rates of agriculture fertilizers and biofertilizers can benefit plant development and nutrient uptake (Adesemoye et al., 2010; Duarah et al., 2011; Mäder et al., 2011). PGP *Pseudomonas* strains have been shown to have the ability to induce disease resistance in pearl millet as well as increase biomass under greenhouse and laboratory conditions (Jogaiah et al., 2010). In a study by Babu et al. (2015), rhizobacteria were reported to improve growth and fruit weight in tomato as well as protection against early blight disease (*Alternaria solani*). Some of our strains have biocontrol properties *in vitro* but they were not evaluated for biocontrol activity as part of this study, however, disease pressure was not observed during the timeline of the field experiment (note a commercial fungicide was applied at week 40).

## Conclusion

Greenhouse and field trials were carried out using individual endophytic *P. fluorescens* strains and an endophytic microbial consortium. In greenhouse experiments, individual strains tagged with gfp and Km^r^ were shown to effectively colonize the rhizosphere and root of *B. napus* (but not the leaves) and they demonstrated a significant increase in plant biomass compared to the non-inoculated control. In the field experiment analysis, the data provide evidence that the application of the live bacterial biofertilizers could enhance aspects of crop development in *B. napus* at field scale. The field data demonstrated statistically significant increases in crop height, stem/leaf, and pod biomass, particularly, in the case of the consortium inoculated treatment. Due to the agronomic conditions pertaining to the experiment (normal fertilizer, pesticide, and fungicide application) the most likely traits implicated in the observed effect were ACC deaminase activity and IAA synthesis. Seed and oil yield were increased in the field in response to inoculation, and although these data were not statistically significant, a real economic value to the grower could be envisaged.

Future work will address the optimization of their application with reduced synthetic fertilizer use with a view to the maximization of crop growth and yield potential. Overall focus, based on this work, should be directed at combining PGP strains with efficient plant colonization ability and that express a range of PGP traits, for use as consortia applications on globally important crops.

## Dedication

We would like to dedicate this work to the family and friends of Dr. Paul Galbally who sadly passed away during the preparation of this paper.

## Author Contributions

DR, KG, JS, and DD conceived of and designed the study. RL, PG, and AM designed and performed the greenhouse and field experiments. RL, KG, and DD wrote the paper.

## Conflict of Interest Statement

The authors declare that the research was conducted in the absence of any commercial or financial relationships that could be construed as a potential conflict of interest.
