# Supplementary material for: Application of Endophytic Pseudomonas fluorescens and a Bacterial Consortium to Brassica napus Can Increase Plant Height and Biomass under Greenhouse and Field Conditions
Source: Front Plant Sci. 2017 Dec 22;8:2193. doi: 10.3389/fpls.2017.02193 (PMC5744461; doi:10.3389/fpls.2017.02193)
Supplement: Supplementary file 1 [file Image_1.pdf]

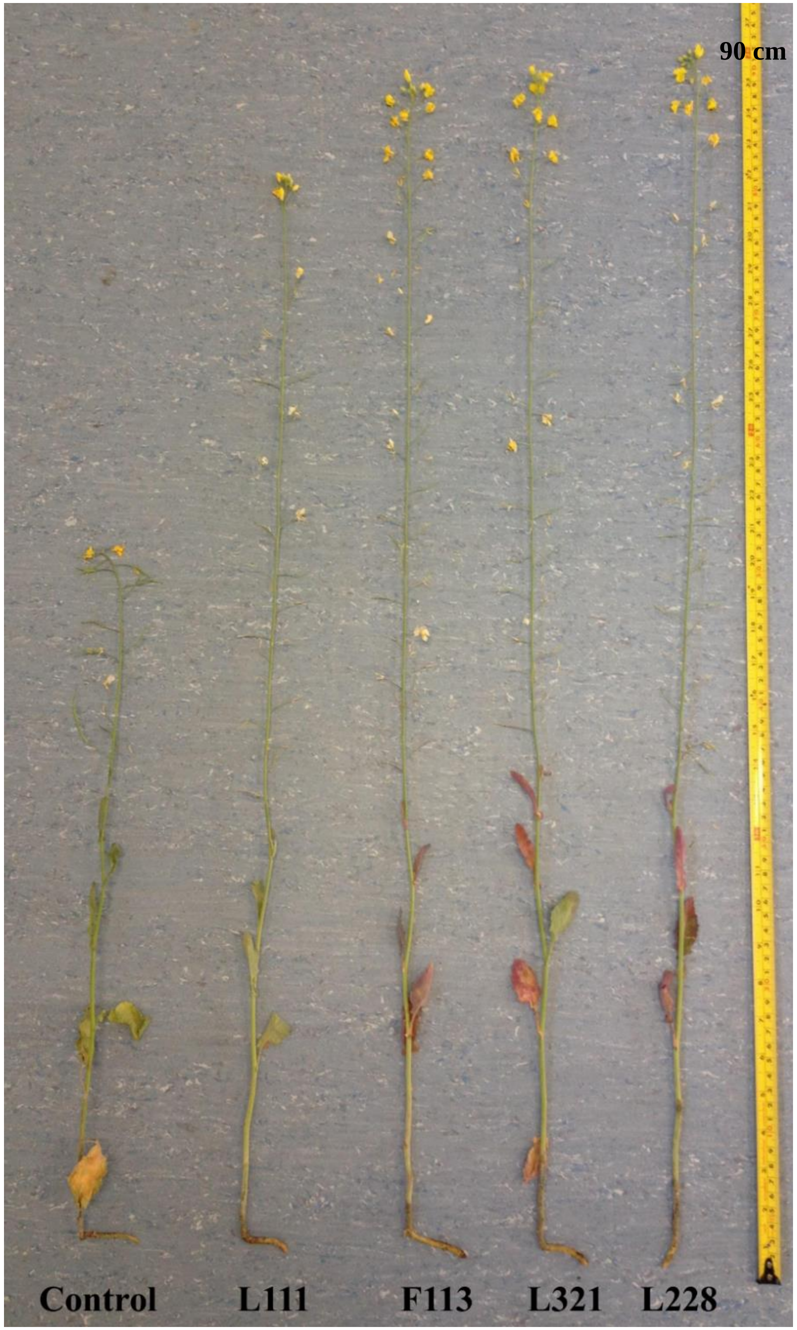

Supplementary Image 1: Length and size differences in *Brassica napus* following treatments of PGPB in the greenhouse experiment.
