# Supplementary material for: Application of Endophytic Pseudomonas fluorescens and a Bacterial Consortium to Brassica napus Can Increase Plant Height and Biomass under Greenhouse and Field Conditions
Source: Front Plant Sci. 2017 Dec 22;8:2193. doi: 10.3389/fpls.2017.02193 (PMC5744461; doi:10.3389/fpls.2017.02193)
Supplement: Supplementary file 2 [file Image_2.pdf]

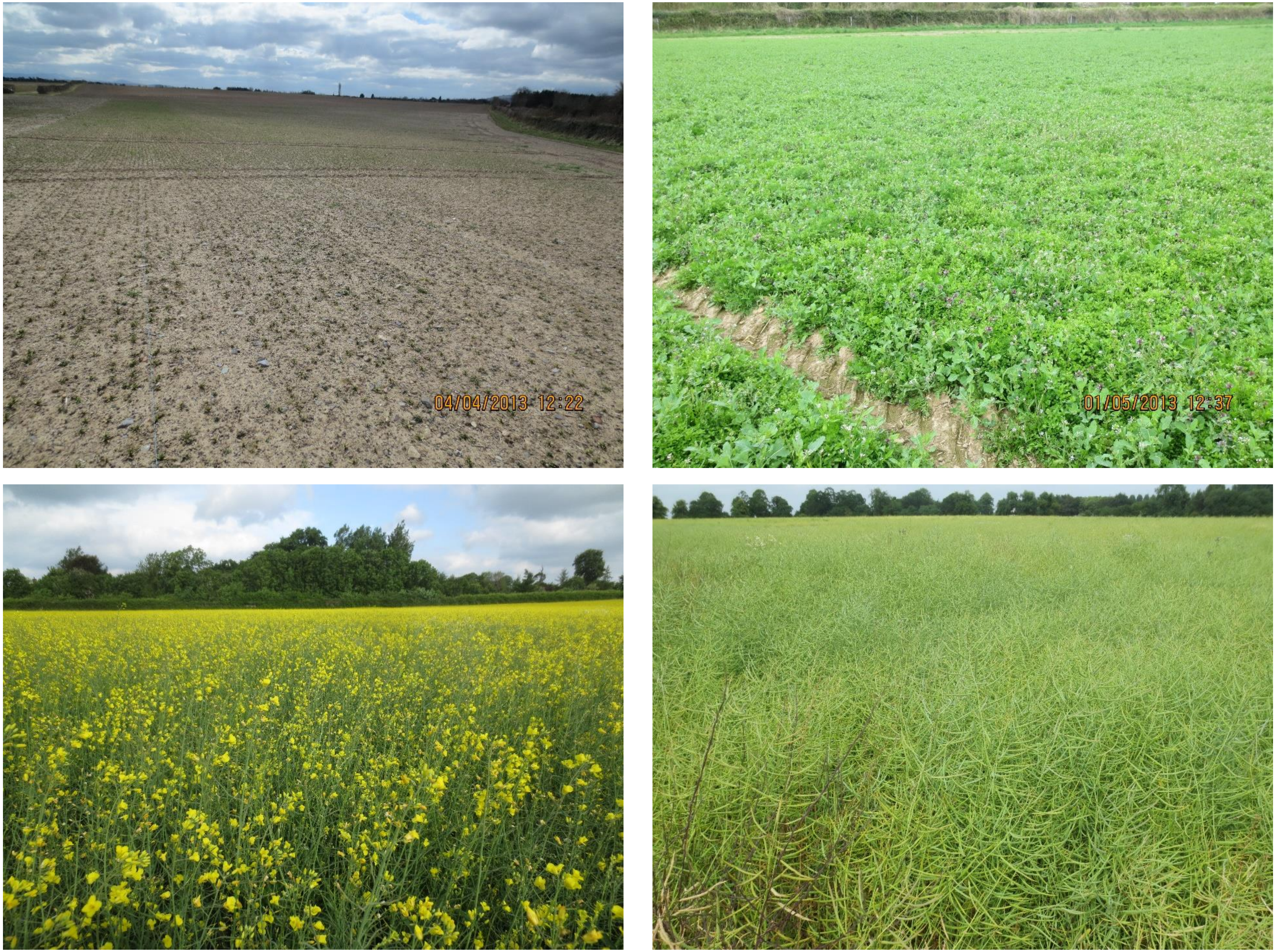

Supplementary Image 2: Field trial images obtained throughout crop lifecycle. Images represent the crop during early development, during the flowering phase and at full pod development.
