# Supplementary material for: Application of Endophytic Pseudomonas fluorescens and a Bacterial Consortium to Brassica napus Can Increase Plant Height and Biomass under Greenhouse and Field Conditions
Source: Front Plant Sci. 2017 Dec 22;8:2193. doi: 10.3389/fpls.2017.02193 (PMC5744461; doi:10.3389/fpls.2017.02193)
Supplement: Supplementary file 4 [file Image_4.pdf]

Supplementary Image 4

Colonization of *B.napus* in Greenhouse experiments : epifluorescent micrographs of L321 (A,C) and plant length after 24 weeks.

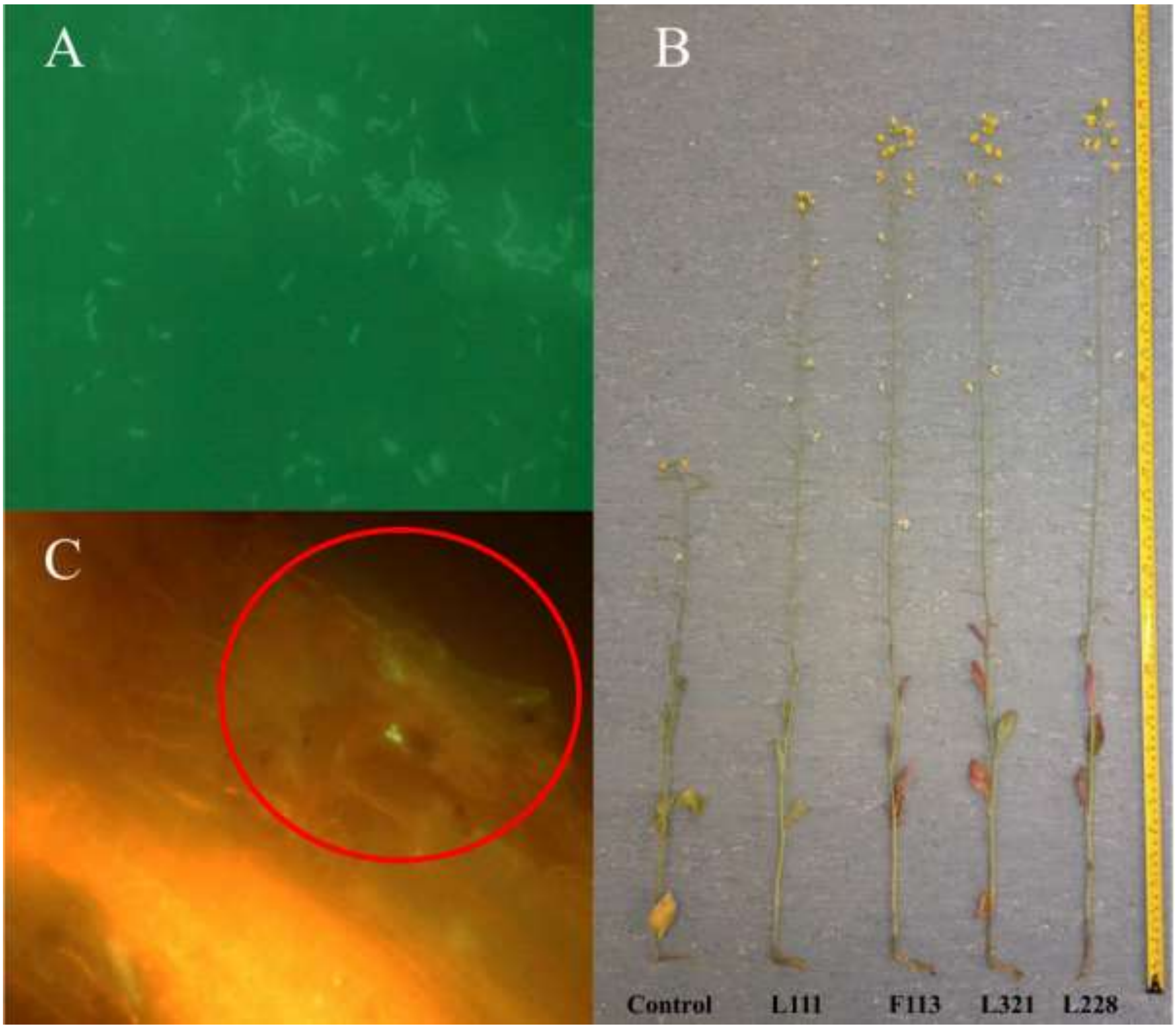

Images taken during the greenhouse trial. A) Confirmation testing. An image of the of the *gfp*:*Kan*^{r} bacteria after isolation on selective agar, under UV light. B) Plant height after 24 weeks under Greenhouse conditions. C) Epifluorescence imaging. This image shows a micro colony of L321 colonising the surface tissue of a *B. napus* root (red circle) at 24 weeks. Microscopy images A and C were taken at a total magnification of 1000x.
